# A role for adverse childhood experiences and depression in preeclampsia

**DOI:** 10.1017/cts.2023.704

**Published:** 2024-01-12

**Authors:** Monica Myers, Serena Gumusoglu, Debra Brandt, Amy Stroud, Stephen K. Hunter, Julie Vignato, Virginia Nuckols, Gary L. Pierce, Mark K. Santillan, Donna A. Santillan

**Affiliations:** 1 Department of Obstetrics & Gynecology, University of Iowa, Iowa City, USA; 2 Iowa Neuroscience Institute, Iowa City, USA; 3 Department of Psychiatry, University of Iowa, Iowa City, USA; 4 College of Nursing, University of Iowa, Iowa City, USA; 5 Department of Health and Human Physiology, University of Iowa, Iowa City, USA; 6 Department of Internal Medicine, University of Iowa, Iowa City, USA

**Keywords:** Preeclampsia, depression, adverse childhood experiences, trauma, neglect

## Abstract

**Introduction:**

Adverse childhood experiences (ACEs) are a measure of childhood adversity and are associated with life-long morbidity. The impacts of ACEs on peripartum health including preeclampsia, a common and dangerous hypertensive disorder of pregnancy, remain unclear, however. Therefore, we aimed to determine ACE association with peripartum psychiatric health and prevalence of preeclampsia using a case–control design.

**Methods:**

Clinical data were aggregated and validated using a large, intergenerational knowledgebase developed at our institution. Depression symptoms were measured by standard clinical screeners: the Patient Health Questionnaire-9 (PHQ-9) and the Edinburgh Postnatal Depression Scale (EPDS). ACEs were assessed via survey. Scores were compared between participants with (*N* = 32) and without (*N* = 46) prior preeclampsia.

**Results:**

Participants with ACE scores ≥4 had significantly greater odds of preeclampsia than those with scores ≤ 3 (adjusted odds ratio = 6.71, 95% confidence interval:1.13–40.00; *p* = 0.037). Subsequent speculative analyses revealed that increased odds of preeclampsia may be driven by increased childhood abuse and neglect dimensions of the ACE score. PHQ-9 scores (3.73 vs. 1.86, *p* = 0.03), EPDS scores (6.38 vs. 3.71, *p* = 0.01), and the incidence of depression (37.5% vs. 23.9%, *p* = 0.05) were significantly higher in participants with a history of preeclampsia versus controls.

**Conclusions:**

Childhood sets the stage for life-long health. Our findings suggest that ACEs may be a risk factor for preeclampsia and depression, uniting the developmental origins of psychiatric and obstetric risk.

## Introduction

Adversity in early childhood is one critical arbiter of life-long health of both adults and their offspring. An emerging literature has revealed links between early-life adversity and adult health in pregnant and nonpregnant populations, including between childhood adversity and adult metabolics, cardiovascular risk, and obstetric disease. For instance, parental separation/divorce, physical and emotional neglect, and psychological abuse in childhood are associated with decreased high-density lipoprotein cholesterol at mid-life, while parental neglect and offending are associated with increased triglycerides and glycated hemoglobin, respectively, in adulthood [1]. However, studies that examine composite measures of childhood adversity across multiple domains of experience are necessary to reveal links between these early-life exposures and life-long medical outcomes with intergenerational implications.

The Adverse Childhood Experiences (ACEs) Questionnaire was developed as a composite self-report measure of abuse, neglect, and household challenges in childhood (before the age of 18). High ACE counts are predictive of long-term cardiometabolic disease. Half of the leading causes of death are associated with ACEs [2]. The effects of ACEs may be additive, compounding with other developmental stressors (e.g., racism, sexism, medical comorbidities, etc.) to increase risk for health conditions across the lifespan. ACEs are also passed trans-generationally, with high numbers of parental ACEs adversely impacting outcomes including child physical and psychological problems, temperament, academic performance, behavioral problems, and infant/toddler development [3]. Understanding ACE impacts on pregnancy health and well-being is therefore a critical step in revealing mechanisms by which these adverse experiences program the health of birthing people and their children.

Epidemiological studies demonstrate a link between childhood adversity measured via ACE scores and adult development of cardiovascular and obstetric disease. For instance, population studies report associations between high numbers of ACEs (≥4) and adult stroke (odds ratio (OR): 2.4, 95% confidence interval (CI): 1.3–4.3) and ischemic heart disease (OR: 2.2, 95% CI: 1.3–3.7) [2]. Importantly, depression may mediate these impacts in part because depression itself is linked with increased ACEs and with cardiovascular disease risk [4]. This link between ACEs and cardiovascular disease is supported by clear molecular mechanisms—dysregulated endothelin-1 and leptin are both associated with both ACEs and cardiovascular disease [5].

Pregnancy is a time of particular vulnerability for mental health and cardiovascular disease risk, possibly owing to the high metabolic, immune, and physiological demands and changes involved with accommodating the feto-placental unit [6– 8]. Preeclampsia is a pregnancy-onset hypertensive disease which occurs in 5%–10% of all pregnancies and increases risk for mood disorders and perinatal morbidity and mortality [9,10]. Common origins for both preeclampsia and mood disorders may be developmental and rely on conserved pathoetiologic mechanisms including stress reactivity and proinflammation [11–13]. ACEs, which developmentally precede pregnancy-onset disease and mood disorders, are known drivers of stress reactivity and inflammatory dysregulation and may serve as a modifiable risk factor in psycho-obstetric disease pathogenesis [14– 17]. Despite this, few previous reports have evaluated the potential links between ACEs and perinatal health, leaving the early-life origins of and interactions with depression, anxiety, and cardiovascular disease risk in the perinatal period largely unexplored. The existing prior work is limited by cohorts under-enriched for those with high ACE scores or without recorded ACEs at all [18].

In the present study, we assessed whether there is an increased rate of ACEs and peripartum depression among participants with preeclampsia versus those without preeclampsia. Depression during pregnancy (prior to preeclampsia diagnosis) and in postpartum were assessed by the Patient Health Questionnaire-9 (PHQ-9) and The Edinburgh Postnatal Depression Scale (EPDS), respectively. We also evaluated classes of ACEs to determine whether abuse, neglect, and/or household challenges in childhood were particularly associated with the emergence of preeclampsia in pregnancy. We hypothesized that participants with preeclampsia would be more likely to have a history of ACEs and increased peripartum depression.

## Materials and methods

### Approvals

This study was approved by the University of Iowa Institutional Review Board as part of the Mechanisms of Early and Late Postpartum Hypertension in Human Preeclampsia study (IRB# 201808705). This study conforms to the US Federal Policy for the Protection of Human Subjects.

### Cohort design and data extraction

Cohorts were designed from the Iowa Intergenerational Health Knowledgebase (IHK, formerly called the Maternal Child Knowledgebase, IRB#202101369), a large clinical knowledgebase which securely collates and integrates information from the electronic medical record on patient diagnoses, demographics, vitals, screenings, and other medical information [19]. Patients were anonymized by a third-party before study analyses. Participants were recruited from a large midwestern academic research hospital by direct recruitment of potential participants during their first trimester.

This is a case control study with two study groups: cases diagnosed with preeclampsia during pregnancy and controls not diagnosed with preeclampsia during pregnancy. Any existing medical or psychiatric diagnoses were recorded prior to delivery. Preeclampsia diagnoses were made in the third trimester and conformed to ACOG guidelines [20]. Diagnoses were confirmed by a board-certified Maternal Fetal Medicine OB/Gyn specialist.

Case (*N* = 32) and control (*N* = 46) demographics, anthropometrics, and medical history are described in Table 1. Participants were included if they had delivered a baby within 9–48 months of study data extraction, were between 18 and 46 years old, were in stable health, and were not pregnant at the time of study inclusion. Participants were excluded if they did not meet the inclusion criteria or were incarcerated at the time of study inclusion.

**Table 1.** Comparison of maternal demographics, anthropometrics, and medical history between cohorts. Body mass index (BMI), millimeters of mercury (mmHg)

	Women with preeclampsia (*n* = 32)	Women without preeclampsia (*n* = 46)	*P*-value
Race, white (%)	96.9	95.7	0.39
Ethnicity, non-hispanic (%)	93.8	100	0.04*
Weight at study visit (lbs.)	173.3	160.4	0.10
Average BMI at study visit (kg/m^2^)	30.0	26.3	0.02*
Participants reporting no chronic conditions (%)	18.8	41.3	0.05*
Participants reporting never smoking (%)	62.5	76.1	0.11
Average time postpartum at study visit (years)	1.64	1.63	0.47
Mean systolic blood pressure at study visit (mmHg)	120.1	110.5	0.0003*
Mean diastolic blood pressure at study visit (mmHg)	68.0	61.0	0.0003*

* Significant by *t* test or chi-square test.

### ACE questionnaire

Between one and two years (average 1.6 years) postpartum, participants completed an ACE questionnaire, which is a validated, ten-item self-report instrument that asks participants about specific adverse experiences they have experienced before the age of 18 [2]. This includes experience across three domains: abuse (physical, emotional, and sexual), neglect (abuse, physical, and emotional), and household challenges (parental divorce or separation, domestic violence, alcohol or drug abuse, mental illness or suicide, and incarcerated persons in the family or home). ACE scores are calculated by summing affirmative responses. Score totals range from 0 to 10, with higher scores indicating greater numbers of adverse experiences. Individuals with ACE scores of 4 or greater are considered “high risk” for a variety of health conditions including depression [21], asthma [22], sleep disturbances [23], chronic pain [24], headaches [25], mortality, heart disease, and dementia risk [26]. Given this, high ACE scores were defined as ≥4.

### EPDS and PHQ-9 scores

The EPDS [27] and Patient Health Questionnaire-9 (PHQ-9) [28,29] were used to evaluate postpartum depression (PPD) and antepartum depression symptoms, respectively. PHQ-9 scores were extracted from the medical record prior to preeclampsia diagnosis in the third trimester, while EPDS scores were extracted from postpartum visits. The EPDS and PHQ-9 are administered as part of routine clinical care.

EPDS scores were stratified as: 0–6 as none or PPD, 7–13 as mild PPD, 14–19 as moderate PPD, and 20–30 as severe PPD. PHQ-9 scores were stratified as follows: 0–4 as none or minimal depression, 5–9 as mild depression, and 10–14 as moderate depression.

### Blood pressure assessments

Between one and two years (average 1.6) after delivery at a study visit, three automated blood pressure readings were acquired by a trained experimenter at one-minute intervals following 10 minutes of seated rest and averaged (Microlife BP3GU1-8X; Clearwater, FL, USA).

### Analyses

To test feasibility of the hypothesis that participants with preeclampsia have higher average ACE scores than those without preeclampsia, we performed a preliminary and speculative one-tailed *t* test. This was followed by a multiple logistic regression (SigmaPlot for Windows version 14.5), to test the association between ACE scores and preeclampsia risk via co-variables. Adjusted odds ratio (aOR) was calculated accounting for known co-variables of interest and with impacts on preeclampsia risk and/or known to interact with ACE scores: overweight (BMI >25) [30], presence of preeclampsia medical risk factors (diabetes, hypertension, heart disease, autoimmune disorders, and/or kidney or bladder condition) [31,32], lifetime smoking status and exposure [33], race/ethnicity (Hispanic or Latino, multiple race, Black, White, Asian) [34], any psychiatric diagnosis (e.g., anxiety, depression, ADHD, etc.) [35], and advanced maternal age (>35 years of age) [36].

Differences between groups were evaluated by Chi-square or student’s *t* test, correcting for multiple comparisons as appropriate. Odds ratio was calculated by contingency table and *p* value by Fisher’s exact test.

*P* < 0.05 was considered statistically significant for all tests. Plots depict means and standard error of the mean (SEM), unless otherwise indicated. Figures were generated using GraphPad Prism (version 9.4.1, GraphPad Software) and Excel (version 2202, Microsoft).

## Results

Seventy-eight participants were included in this study and provided complete ACE questionnaires: 32 with a history of preeclampsia and 46 without prior preeclampsia. Groups were not statistically different by race, body weight, smoking history, or length of time postpartum. However, the preeclampsia group was statistically more likely than controls to be Hispanic, had higher BMIs, more chronic conditions such as hypertension and diabetes, and had higher systolic and diastolic blood pressures (Table 1).

### ACEs and preeclampsia

**Figure 1.**
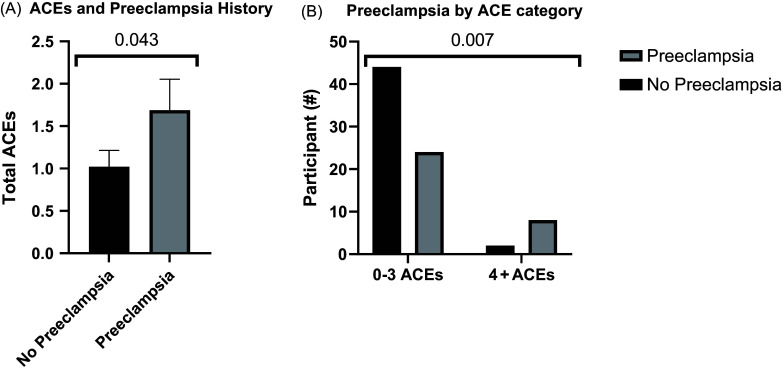
Increased adverse childhood experiences (ACEs) are associated with a history of preeclampsia. P value by one-tailed *t* test (A) and P value by two-sided chi-square (B).

As hypothesized, participants with prior preeclampsia (*N* = 32) had significantly higher average ACE scores than controls (*N* = 46) (1.69 vs 1.02, *p* = 0.043) via an exploratory, one-tailed *t* test (Fig. 1A). Participants were binned according to their number of ACEs, with 4 or more ACEs considered high risk, as outlined in the Materials and Methods section. Further exploratory analyses revealed that participants with ACE scores ≥ 4 (*N* = 10) more often had preeclampsia than those with ACE scores ≤ 3 (*N* = 68) (80.0% vs 35.3%, *p* = 0.007 by chi-square) (Fig. 1B).

To test the relationship between preeclampsia and exposure to ACEs in a more comprehensive way, we next performed a multivariate regression. When adjusted for multiple covariates (BMI, maternal age, diagnosis associated with preeclampsia risk, lifetime smoking status, race/ethnicity, and psychiatric diagnosis), the odds of having preeclampsia was significantly increased in participants with ≥4 ACEs as compared to those with ≤ 3 ACEs (aOR = 6.71; 95% CI = 1.13, 40.00; *p* = 0.037). Of those covariates tested, only a diagnosis associated with preeclampsia risk (diabetes, hypertension, heart disease, autoimmune disorders, and/or kidney or bladder condition) was also significantly associated with increased preeclampsia (aOR = 5.51; 95% CI = 1.78, 17.11; *p* = 0.003) (Table 2).

**Table 2.** Multiple logistic regression analysis of relationship between adverse childhood experiences (ACEs) and preeclampsia, covarying for maternal body mass index (BMI), smoking history, race/ethnicity, psychiatric conditions, medical conditions, and advanced maternal age. Confidence interval (CI)

Variable	Coefficient (*β*)	Standard error	Wald statistic	*P*	Odds ratio	95% CI
Intercept	−1.19	1.39				
ACEs (≥4)	1.90	0.91	4.37	*0.037*	6.71	1.13–40
BMI (≥25, overweight)	0.44	0.57	0.59	0.442	1.55	0.51–4.71
Ever smoker	−0.17	0.63	0.07	0.791	0.85	0.25–2.89
Race/Ethnicity	−0.45	1.36	0.11	0.740	0.64	0.04–9.19
Psychiatric condition	1.01	0.60	2.78	0.095	2.74	0.84–8.92
Medical condition*	1.71	0.58	8.73	*0.003*	5.51	1.78–17.11
Advanced maternal age (≥35)	−0.78	0.78	0.99	0.320	0.46	0.01-2.13

* Medical conditions associated with increased risk for preeclampsia were included: diabetes, hypertension, heart disease, autoimmune disorders, and/or kidney or bladder condition.

**Figure 2.**
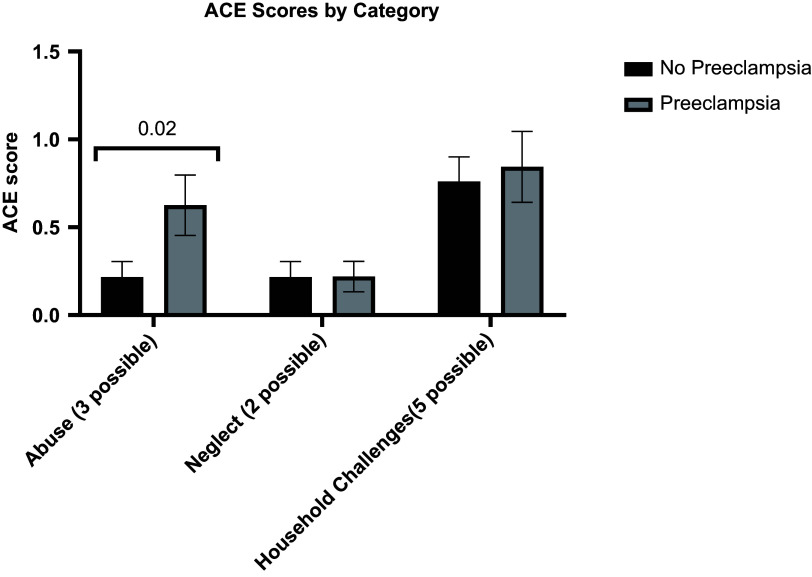
Adverse childhood experiences (ACE) scores in participants with and without preeclampsia, subset by ACE category. *P < 0.05 by two-tailed *t*-test with correction for multiple comparisons via the Bonferroni–Dunn method.

Subsequent, exploratory tests examined which domain(s) of ACEs (trauma, abuse, and household challenges) might have contributed to increased preeclampsia. Women with a history of preeclampsia were significantly more likely to respond affirmatively to items on the ACE questionnaire about abuse (*p* = 0.02) than control women, while the number of household challenge and neglect ACEs was not different between groups (Fig. 2).

### Preeclampsia and PHQ-9 and EPDS scores

**Figure 3.**
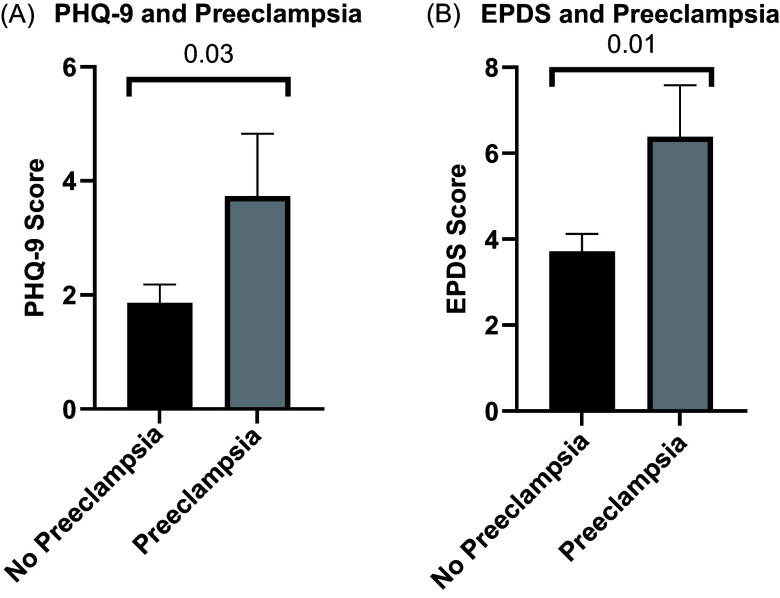
Average Patient Health Questionnaire-9 (PHQ-9) and the Edinburgh Postnatal Depression Scale (EPDS) scores are increased in participants with a history of preeclampsia. P values by two-tailed t tests.

PHQ-9 scores and EPDS scores were available for the subset of participants cared for throughout their pregnancy and postpartum at our institution. Among the 52 participants with available PHQ-9 scores, women with history of preeclampsia (*N* = 15) had higher average PHQ-9 scores than those without a history of preeclampsia (*N* = 37) (3.73 ± 4.23 vs. 1.87 ± 1.95, *p* = 0.03) (Fig. 3A) (Table 3). Similarly, among the 63 participants with available EPDS scores, participants with a history of preeclampsia (*N* = 21) had higher average EPDS scores than those without a history of preeclampsia (*N* = 42) (6.38 ± 5.51 vs. 3.71 ± 2.64, *p* = 0.01) (Fig. 3B) (Table 3).

**Table 3.** Edinburgh Postnatal Depression Scale (EPDS) and Patient Health Questionnaire (PHQ-9) scores and depression rates in participants with and without preeclampsia

	Preeclampsia	No preeclampsia	*P*-value
Diagnosed depression (%)	37.5 (*n* = 32)	23.9 (*n* = 46)	0.05*
Average PHQ-9 score	3.73 ± 4.23 (*n* = 15)	1.86 ± 1.95 (*n* = 37)	0.03*
Average EPDS score	6.38 ± 5.51 (*n* = 21)	3.71 ± 2.64 (*n* = 42)	0.01*

* Significant by *t* test or chi-square test. Mean ± standard deviation.

### ACEs and depression

**Figure 4.**
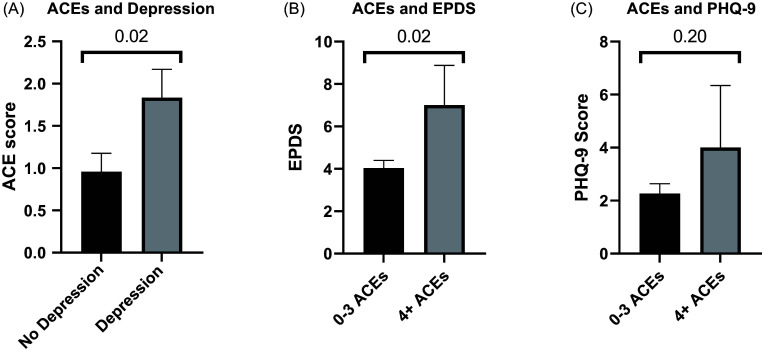
Adverse childhood experiences (ACEs) are increased with depression, and those with 4 or more ACEs have increased Edinburgh Postnatal Depression Scale (EPDS) and Patient Health Questionnaire-9 (PHQ-9) scores relative to those with 0-3 ACEs. P values by two-tailed *t* tests.

Among the 78 participants, 30 had a documented diagnosis of depression. Additional exploratory analyses revealed that participants with depression had higher average ACE scores than those without depression (1.83 vs. 0.96, *p* = 0.02) (Fig. 4A).

Women with ACE scores ≥ 4 had significantly higher average EPDS scores than those with ACE scores ≤ 3 (7.00 vs. 4.04, *p* = 0.02) (Fig. 4B). PHQ-9 scores did not differ significantly between those with ACE scores ≤ 3 and those with scores ≥ 4 (2.27 vs. 4.00, *p* = 0.20) (Fig. 4C).

## Discussion

ACEs are a common early-life exposure that may program pregnancy, postpartum, and intergenerational health. The landmark Adverse Childhood Experiences Study reports that more than half of US adults have experienced greater than 1 category of ACEs, with 6.2% reporting four or more exposures [2]. In individuals with high numbers of ACEs (≥4), we find an increased prevalence of preeclampsia, even after controlling for psychiatric and demographic covariates. Stressors in the postpartum period may particularly aggravate underlying vulnerability, as postpartum EPDS but not intrapartum PHQ-9 scores were increased in those with high numbers of ACEs via additional, exploratory analyses. Furthermore, as others have shown [4,37–40], we find significant comorbidity between preeclampsia and depression, with those with a history of preeclampsia having a significantly increased incidence of depression. We propose that ACEs may represent a developmental origin point for psycho-obstetric risk; increased depression and preeclampsia risk are bidirectionally associated with one another in adults [11,41–45]. Intergenerational programming of child health risk (e.g., for neurodevelopmental/behavioral, metabolic disorders) by high parental ACEs may occur via multiple mechanisms, including molecular placenta–brain axis mechanisms (e.g., extracellular vesicles, hormones, inflammatory factors) [46–50], epigenetic mechanisms (e.g., in maternal mitochondria, paternal sperm) [51], psychosocial risks and exposures (e.g., socioeconomics, pollution) [52,53], and community or sociopolitical dynamics (e.g., community violence, racism) [54].

Vascular, inflammatory, and stress reactivity mechanisms may be programmed by ACE exposures in early life [55–57]. These same mechanisms are perturbed in both mood disorders and in preeclampsia [11,39,58], thereby potentially increasing psycho-obstetric risk in adulthood. Depression may link ACEs to preeclampsia indirectly, or via more direct mechanisms such as increased epicardial adiposity, interference in blood pressure control by antidepressive agents, or by other mechanisms [32,45,59]. Revealing these conserved risk mechanisms is of critical relevance to developing high-yield therapeutic and prophylactic approaches. For instance, an emerging literature indicates that selective serotonin reuptake inhibitors (SSRIs) may mediate broader mechanisms, such as inflammatory CD4+ T cell reactivity and vascular/platelet reactivity, which underlie both depression and preeclampsia pathogenesis [44,45].

The use of ACEs as a measure of early-life environmental exposures is confounded by genetic and environmental factors. In particular, several experiences included in the “household challenge” ACE category are attributable to genetic factors, including mental illness (depression, suicidality) and substance abuse in the home environment. We evaluated these impacts by speculatively analyzing ACE categories separately and found that the abuse category significantly contributed to preeclampsia risk while household challenges and neglect categories did not. It remains important to consider that ACEs are not purely a measure of environmental risk, but also incorporate genetic risk. While we were underpowered here to build a full model accounting for possible confounds, future studies will need to address various contributions and interactions between genetic and environmental components in early life in the programming of psycho-obstetric risk. Despite these limitations, our results indicate that ACEs are a useful clinical heuristic, which can inform clinical decision-making and risk stratification.

While some confounds (maternal age, weight, smoking status, race/ethnicity, and psychiatric and medical comorbidities) were dealt with utilizing multiple logistic regression, others remain which are important to consider given interacting impacts on ACEs and preeclampsia. For instance, socioeconomic status was not evaluated here as data on household income were not accessible. Socioeconomics have profound effects on health outcomes and interact with the impacts of early-life trauma and stressors on cardiovascular, metabolic, and pregnancy-related outcomes [14,54,60].

While highly standardized, the ACE questionnaire used here also does not provide a comprehensive view of all forms of childhood adversity. For example, trauma related to socioeconomic or racial/ethnic discrimination are not considered. We were limited in our ability to incorporate socioeconomic measures such as family income in our analyses. Socioeconomic status (SES) interacts with ACEs, and prior work has found that ACE-associated risk for cognitive health is mediated by childhood SES in white but not Black participants [60]. Our sample was also homogeneous and limited by low levels of racial and ethnic diversity. It will be important for future work to address impacts of ACEs on obstetric risk in minoritized populations. Prior work finds, for example, that increased ACEs among American Indian women are associated with decreased prenatal care relative to white women, which may be an important factor in peripartum disease diagnosis and treatment disparities in this population [61]. Future work should incorporate SES, employment status, and other socioeconomic stressors, as well as more diverse samples into our measures of obstetric disease outcomes.

Larger sample sizes will allow for additional granularity of these analyses. Multistate and national cohorts must have ACE measures integrated to better capture the impacts of early-life adversity on outcomes including pregnancy-related morbidity. Additionally, it is imperative that longitudinal cohorts be established to follow individuals and further associate early-life experiences and exposures with depression and subsequently with gestational morbidity. In concert with thoughtful preclinical work (e.g., animal models of depression with preeclampsia-like phenotypes [62]), these longitudinal studies may begin to address the causal links between psychiatric and obstetric disease.

Combinatorial, additive impacts of ACEs and medical and psychiatric disabilities are also not included. For example, ACEs may have different impacts on a child with attention deficit hyperactivity disorder than on a neurotypical child [63]. Furthermore, the mitigating influences of non-parental caregivers, environmental factors, and interpersonal resilience were not considered here. Future studies may examine these elements to determine protective and risk factors in the pathogenesis of psycho-obstetric risk.

Retrospective ACE questionnaires may also be vulnerable to overestimated impact of adversity on outcomes by way of recall bias. On average, nearly 2 years elapsed between patient delivery and completion of the ACE questionnaire in the present study. However, the literature also reports that retrospective ACE records are associated with prospective ones, and that retrospective report bias is not sufficient to invalidate case–control study measures of well-defined adversity, as in the ACE questionnaire [64,65]. Furthermore, studies report that recall of early-life trauma may have more bearing on psychological and medical morbidity risk than do objective rates of exposure [64]. In exploratory work assessing the impact of obstetric outcomes on recall of childhood maltreatment, most participants’ recall of maltreatment agreed between antenatal to postpartum timepoints. Disagreement between antenatal and postpartum recall of childhood maltreatment increased only with regard to childhood physical neglect in those who experienced adverse obstetric outcomes [66]. This recall-related bias may have played less of a role in the present study, as we do not find significant increases in neglect-related ACE scores among preeclamptic versus control pregnancies here.

A further source of bias which should be considered here is selection bias, given that the sample size assessed here was limited to midwestern women who were cared for at a large, academic, tertiary care hospital which is the only one of its kind in the state. Participants in academic studies are notoriously homogenous and often exclude various forms of diversity (racial/ethnic, socioeconomic, language, disability, etc.). Additionally, those who are cared for at the state’s only tertiary care hospital have increased morbidity and medical complexity, furthering possible sample bias here. Community outreach is one strategy to improve diverse recruitment, as is involvement of smaller, primary care hospitals and healthcare centers including those in rural areas.

Our findings suggest that ACEs may predict risk for preeclampsia, even after controlling for psychiatric and other comorbidities and relevant clinical characteristics. These results reveal a potential role for early-life prevention efforts in the mitigation of life-long cardiovascular and psychiatric disease risk. When they occur in pregnancy, these diseases may intergenerationally program poor health outcomes and are therefore high-yield targets for intervention, risk-stratification, and clinical planning.

Given the high incidence of elevated ACEs, preeclampsia, and depression, it is critical that we determine conserved, modifiable developmental mechanisms for therapeutic targeting and prevention. The deleterious impacts of ACEs across the lifespan can be mitigated by increased referral to effective support services, family-centered treatment and parenting interventions, substance abuse treatment, and enhanced school conflict and emotion management supports, among other things. Clinicians may also wish to incorporate ACE measures into their pregnancy planning, prenatal care, and postpartum wellness visits. These measures can be rich and meaningful predictors of psycho-obstetric health. Future work should evaluate the clinical application of these strategies in mitigating life-long pregnancy and psychiatric disease risk in birthing people and their families.
